# From Single Cells to Silicon: Emerging Technologies Transforming Monoclonal Antibody Discovery

**DOI:** 10.3390/antib15030047

**Published:** 2026-05-29

**Authors:** Victoria Sherwood, Denise Harold, Richard O’Kennedy, Christine Loscher, Paul Leonard

**Affiliations:** School of Biotechnology, The DCU Life Sciences Institute, Dublin City University, Glasnevin, D09 V209 Dublin, Ireland; victoria.sherwood2@mail.dcu.ie (V.S.); denise.harold@dcu.ie (D.H.); richard.okennedy@dcu.ie (R.O.); christine.loscher@dcu.ie (C.L.)

**Keywords:** microfluidics, microtools, monoclonal antibody discovery, in silico discovery, single cell analysis, artificial intelligence, machine learning

## Abstract

Monoclonal antibody (mAb) discovery has been transformed by advances in single-cell technologies, microfluidics, high-throughput sequencing, and computational design. Modern platforms enable the interrogation of large numbers of individual B cells, directly linking antibody sequence with antigen specificity and functional activity. Microfluidic and optofluidic systems now support high-throughput compartmentalisation and functional screening of antibody-secreting cells, while sequencing-based approaches allow parallel recovery of paired heavy- and light-chain sequences. These developments have shifted antibody discovery from binding-based selection toward function-first paradigms, enabling the rapid identification of diagnostic and therapeutically relevant antibodies. Integration with computational tools, including machine learning and structure-based modelling, has further enabled the emergence of closed-loop discovery pipelines, in which experimental and in silico methods iteratively refine candidates. This review summarises key advances in single-cell microtools over the last decade and highlights how the convergence of experimental and computational technologies is reshaping antibody discovery toward scalable, data-driven, and increasingly automated platforms.

## 1. Introduction

Monoclonal antibodies (mAbs) have emerged as one of the most transformative classes of therapeutics in modern medicine, fundamentally reshaping the treatment landscape [1,2]. They now represent the largest and fastest-growing class of biotherapeutics, with more than 170 antibody-based therapeutics approved globally, and with several of the top-selling drugs including adalimumab, rituximab, trastuzumab and infliximab belonging to this category [3]. Their excellent binding specificity, favourable safety profiles, and engineering versatility have made them indispensable across therapeutic and diagnostic platforms [1]. The urgency of the COVID-19 pandemic further highlighted the need for rapid and scalable antibody discovery pipelines. This accelerated innovation across both experimental and computational domains.

Despite their clinical and commercial success, the discovery process remains a complex and resource-intensive process. The growth of antibody-based therapeutics has placed increasing pressure on the biopharmaceutical industry to reduce development timelines, improve efficiency, and lower attrition rates [4]. Traditional discovery pipelines are often time-consuming, requiring extensive screening and optimisation before viable candidates are often identified. Historically, antibody discovery has been dominated by well-established experimental workhorse technologies, most notably hybridoma technology and display-based methods such as phage and yeast display. Hybridoma technology, first described by Köhler and Milstein (1975), involves the fusion of antibody-producing cells with immortalised myeloma cells to generate stable monoclonal antibody-producing cell lines [5]. This approach has been instrumental in the development of early diagnostic and therapeutic antibodies. It remains widely used due to its robustness and ability to generate high-affinity antibodies. Phage display, introduced by McCaffery et al., enables the in vitro selection of antibodies from large combinatorial libraries [6]. This facilitates the identification of fully human antibodies and expands accessible sequence diversity. While still the mainstay in many antibody discovery labs, these conventional approaches are associated with important limitations that restrict their effectiveness in the context of modern drug discovery. Hybridoma methods are inherently low-throughput while being labour-intensive. The method involves immunisation, cell fusion and extensive screening [7]. In addition, they are constrained by the host immune system, which may not fully capture the breadth of therapeutically relevant antibody diversity. Display-based methods, offering increased throughput, rely on artificial pairing of heavy and light chains. This can be disruptive to native V_H_/V_L_ pairing and result in suboptimal binding results [8]. These approaches both require multiple rounds of screening and optimisation to achieve the desirable characteristics such as affinity, specificity, and manufacturability [4]. Neither method readily provides insight into the native immune context or functional state of individual B cells. This limits the ability to directly link the genotype to phenotype [7].

A paradigm shift in antibody discovery occurred during the mid 2000s with the emergence of single-cell analysis technologies. These technologies enable direct examination of individual B cells at exceptional resolution [7] and allow for the recovery of naturally paired heavy- and light-chain antibody sequences, preserving the original immune-derived combinations [8,9]. Advances in microfluidics, high-throughput screening and cell-sorting technologies have made it possible to profile thousands to millions of B cells in parallel. These techniques can capture both sequencing and important data such as antigen specificity, biological function and secretion profiles [10]. These approaches allow for a comprehensive and unbiased exploration of immune repertoires, increasing the chance of identifying high-quality antibody candidates [11].

In parallel with advances in experimental techniques, the past decade has witnessed an explosion in the application of computational methods and artificial intelligence (AI) to antibody discovery [4,11]. These approaches have been driven by improvements in machine learning algorithms, the availability of large-scale biological datasets, and increased computational power. AI tools are now applied across multiple stages of the antibody discovery pipeline. At the structural level, deep learning models such as AlphaFold have enabled highly accurate protein structure prediction, including antibody modelling, facilitating rational design, and reducing reliance on experimental methods [12,13]. At the sequence level, machine learning approaches can identify patterns associated with binding affinity, specificity, and developability assessment of physicochemical properties, guiding candidate selection and optimisation [4]. Furthermore, AI-driven tools enable epitope and paratope prediction, improving mechanistic understanding of antibody–antigen interactions, and supporting targeted engineering strategies [12].

**Figure 1:**
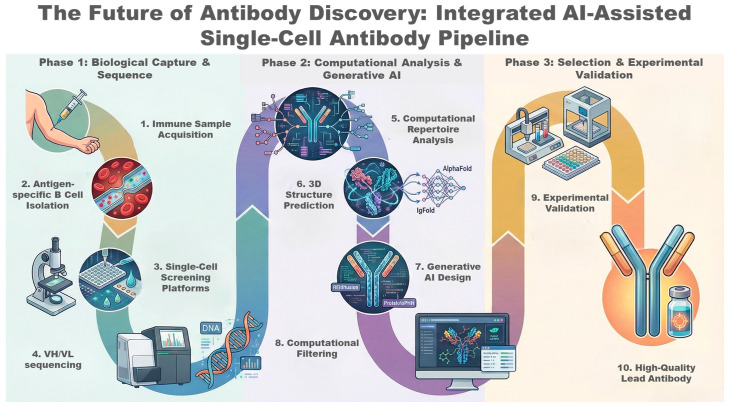
The future of antibody discovery: integrated AI-assisted single-cell antibody pipeline. This figure illustrates a next-generation, closed-loop framework for antibody discovery that integrates single-cell technologies with advanced computational modelling and generative AI. The workflow is organised into three interconnected phases. This review highlights the latest technologies supporting each stage of the pipeline. (**Phase 1**) involves immune sample acquisition, antigen-specific B-cell isolation using platforms such as FACS, microtools and microfluidic systems, followed by high-throughput single-cell screening and paired VH/VL to generate large-scale antibody datasets. (**Phase 2**) uses these datasets for in-depth repertoire analysis, structural modelling and data-driven candidate optimisation. Antibody candidates are improved by generative AI models and prioritised through computational filtering. (**Phase 3**) involves synthesis and experimental validation to confirm the efficacy of the AI-designed antibodies. Resulting in the identification of high-quality antibodies ready for further development.

More recently, generative AI approaches have opened new possibilities for de novo antibody design. These models, including diffusion-based frameworks and protein language models, have the potential to generate entirely novel antibody sequences with desired functional properties, such as enhanced affinity or reduced immunogenicity [14]. By exploring regions of sequence space inaccessible to natural immune responses or traditional library-based methods, generative models are poised to expand the repertoire of candidate therapeutics. Importantly, these computational approaches can be integrated with experimental platforms, enabling iterative design–build–test cycles that accelerate discovery and optimisation, as shown in Figure 1.

While several recent reviews have examined single-cell antibody discovery technologies or AI-assisted computational antibody engineering independently, fewer studies have comprehensively explored the convergence of these approaches within integrated discovery pipelines. In this context, this review explores the integration of single-cell analysis and AI-driven computational tools that reshape antibody discovery. Specifically, we examine (i) the current landscape of single-cell technologies that enable high-throughput isolation and characterisation of antigen-specific B cells; (ii) the in silico and AI-based approaches used to optimise antibody candidates for affinity, specificity and developability and finally; (iii) how wet lab-based confirmation is critical for validation and providing a feedback loop for improving computational approaches. By combining these complementary strategies, next-generation discovery pipelines could overcome the limitations of traditional methods. This will enable faster, more scalable, and precise development of antibodies for diagnostics and therapeutics.

## 2. Phase 1: Biological Capture & Screening

### 2.1. Microengraved Microwells, Chambers and Microcapillary Arrays

**Figure 2:**
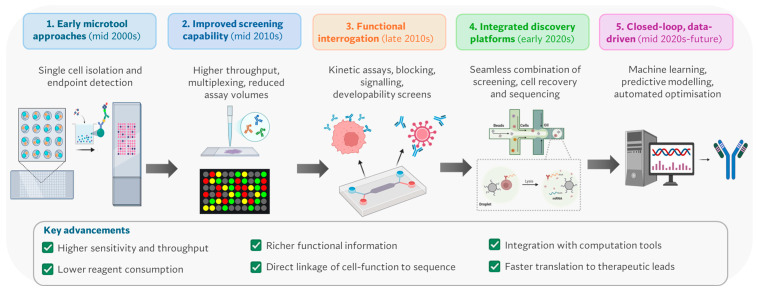
Overview of single-cell analysis technologies evolution over the last decade. The evolution of single-cell microtools has progressed from discrete, low-throughput platforms toward highly integrated systems that enable end-to-end antibody discovery workflows. Early technologies focused on isolating and characterising individual B cells, often requiring separate steps for functional screening, sequence recovery, and analysis. Advances in microfluidics, high-throughput sequencing, and optofluidic platforms have since enabled the parallel interrogation of thousands to millions of cells, directly linking antibody specificity, function, and genotype. More recently, the integration of computational approaches, including machine learning and structure-based modelling, has facilitated the emergence of closed-loop workflows, where experimental data and in silico predictions iteratively inform candidate selection and optimisation. Together, these developments are driving a shift toward scalable, automated, and data-driven antibody discovery pipelines. Icons were generated in BioRender. Leonard, P. (2026) https://BioRender.com/8f11ma7 (accessed on 24 April 2026).

Microwell-based systems isolate individual cells in nanoliter volumes, enabling detection of secreted antibodies via surface capture or in situ fluorescence [15]. Early implementations provided robust single-cell secretion analysis but were limited by endpoint measurements and moderate throughput (Figure 2) [16,17,18]. Recent advances have significantly extended their capabilities. Modern nanoliter-volume well systems now enable multiplexed, time-resolved secretion profiling, allowing dynamic characterisation of antibody production at the single-cell level [19,20]. These platforms support high-content analysis, including secretion kinetics, antigen specificity, and functional phenotyping, transforming microwells into tools for quantitative single-cell immunology rather than simple screening. In addition, microwell platforms are increasingly used in cell line development and productivity screening, linking early discovery with manufacturability considerations [21]. Their strengths lie in spatial addressability, imaging compatibility, and multiplexing flexibility, although throughput remains lower than droplet-based approaches.

Microfluidic chamber-based systems provide precise control over the cellular microenvironment, enabling multi-step assays with reagent exchange, washing, and sequential interrogation [22,23]. This allows functional assays such as affinity measurement, receptor blocking, and signalling modulation to be performed directly at the single-cell level [23,24,25]. Over the last decade, these systems have evolved into advanced optofluidic platforms which enable thousands of individual B cells or plasma cells to be cultured and interrogated in parallel (www.abcellera.com, accessed on 20 April 2026, www.brukercellularanalysis.com/, accessed on 20 April 2026). These platforms allow repeated measurements on the same cell, enabling multiplexed functional profiling, including binding, neutralisation, and developability screening. This has driven a shift toward function-first antibody discovery, where candidates are selected based on biological activity rather than binding affinity alone. Integrated cell export enables recovery of viable cells for downstream sequencing and recombinant expression, preserving native heavy–light chain pairing. Although throughput is lower than droplet systems or microcapillary arrays, microfluidic chambers provide excellent assay flexibility and data richness, making them widely used in therapeutic antibody discovery pipelines.

Microcapillary arrays represent a distinct approach based on fused capillary bundles that enable spatial isolation of millions of cells in extremely small volumes [15,26]. Platforms such as DiCAST demonstrated the ability to screen large populations without enrichment, reducing bias and enabling detection of rare antibody-producing clones. These systems offer exceptional throughput and spatial indexing, as well as multiplexing via removable detection surfaces. They also show versatility in screening antibody-producing cells from both B cells and *E. coli* expressing recombinant antibodies [26]. More recent implementations, such as μSCALE, have found applications in protein engineering and library screening, particularly in microbial systems, but their role in mammalian antibody discovery remains more specialised [27].

### 2.2. Microdroplet and Encapsulation Technologies

Over the last decade (Figure 2), microdroplet systems have moved from proof-of-concept secretion assays to some of the most scalable formats in single-cell antibody discovery. Their core advantage is straightforward: water-in-oil droplets confine one cell with defined reagents in picoliter volumes, so the secreted antibody rapidly accumulates to detectable levels while preserving linkage to the producing cell. Recent reviews [7,28,29] describe droplet workflows as one of the dominant routes for high-throughput discovery because they combine efficient single-cell confinement, low reagent consumption, and compatibility with downstream sorting and sequencing. A major technical advance has been the diversification of what happens inside the droplet. Early droplet assays primarily measured secretion or binding [30]. More recent implementations incorporate capture beads, reporter reagents, paired-cell formats, and controlled reagent addition, allowing screens for antigen binding, blocking activity, or cell–cell interactions [31,32,33,34]. Automated droplet-digital microfluidic platforms now support deterministic encapsulation strategies and repeated washing or reagent exchange steps that were once difficult in classical emulsion workflows, narrowing the gap between droplet screening and more controlled chamber-based assays.

Within this broader class, encapsulation technologies have become especially important. The key innovation here is not simply making droplets but stabilising the microenvironment around the cell so that secreted antibodies are retained locally and can be interrogated more flexibly [35]. DropMap is a representative example that uses picoliter droplets and fluorescence relocation to quantify secretion kinetics and affinity-related behaviour from tens of thousands of individual cells, demonstrating that droplets can support more than yes/no secretion calls [36,37]. This type of platform helped establish that antibody-secreting cells are highly heterogeneous in secretion rate and affinity, and that this heterogeneity can be measured directly rather than inferred after bulk culture. The newest generation of encapsulation systems goes further by combining droplet formation with hydrogel capture matrices making antibody-secreting cells (ASCs), especially plasmablasts and plasma cells, much more accessible for rapid discovery, with reported workflows capable of screening millions of ASCs and generating high-affinity SARS-CoV-2 antibodies in about two weeks [35].

Taken together, the literature now shows that microdroplet and encapsulation technologies are no longer best viewed as simple miniaturised secretion assays. They have become a family of platforms spanning ultrahigh-throughput primary screening, quantitative secretion phenotyping, and increasingly sequence-ready recovery.

### 2.3. Single-Cell Sequencing and Immune Repertoire Profiling

The integration of single-cell sequencing into antibody discovery has fundamentally changed what a “screen” can deliver. Instead of ending with a positive well, droplet, or chamber, modern workflows increasingly end with a paired heavy- and light-chain sequence, a clonotype assignment, and often a linked specificity or functional label. Reviews from the last five years [7,28,29,38,39] consistently identify this shift as one of the major turning points in the field, because it converts single-cell screening from a clone-picking exercise into a data-rich view of the immune repertoire. An example development in this space is LIBRA-seq [40], which links the B cell receptor sequence to antigen specificity by exposing B cells to DNA-barcoded antigens and recovering both antigen barcodes and paired B cell receptors sequences through single-cell sequencing. This was important for two reasons. First, it enabled specificity mapping at much larger scale than conventional one-cell/one-antigen workflows. Second, it made multiplexed antigen profiling practical, allowing investigators to discover not just binders, but cross-reactive or broadly neutralising candidates across antigen panels. Since its introduction, LIBRA-seq and related barcoding strategies have been applied to HIV [40], influenza [41], SARS-CoV-2 [42], and other systems, helping to establish sequencing-assisted antibody discovery as a routine strategy rather than a specialist method. A second important development is the extension of sequencing-based specificity mapping beyond memory B cells to antibody-secreting cells. TRAPnSeq is another example that captures locally secreted immunoglobulins from antibody-secreting cells and couples antigen specificity to sequencing, making it possible to profile antigen-specific IgG- and IgE-secreting cells from mice and humans, addressing a key blind spot in classic BCR-seq workflows by identifying cells that are functionally important because they are actively secreting antibodies but are harder to interrogate by standard antigen-bait sorting alone [43]. More broadly, single-cell immune repertoire profiling is now being used to reconstruct clonal expansion, somatic hypermutation, class switching, and lineage relationships across very large B cell datasets [44]. Recent studies [45,46] combining single-cell transcriptomes with paired repertoire data have shown that clonally expanded and antigen-specific B cells occupy discernible but overlapping transcriptional states, underscoring both the power and the current limits of computational prioritisation from sequence or transcriptome alone. These datasets are increasingly valuable not only for discovering antibodies directly, but also for training predictive models of antigen specificity and developability. The field has, therefore, moved beyond the idea that sequencing is merely a downstream identification tool. It is now a co-equal discovery modality. Single-cell sequencing and immune repertoire profiling provide the molecular scaffold upon which modern discovery is built. They preserve native pairing, reveal population structure, identify convergent responses, and increasingly allow experimental phenotypes to be linked directly to sequence features. In that sense, sequencing has become both a readout and a design engine for the next generation of antibody discovery workflows, especially those incorporating in silico and AI-powered approaches as discussed in the next section.

### 2.4. Scalability, Integration and Throughput

One of the clearest themes in the literature from the last decade is that the field no longer evaluates platforms solely by sensitivity or assay elegance, but by how well they scale and how completely they integrate upstream and downstream steps. The practical benchmark has shifted from “can this platform identify a rare positive?” to “can it identify that positive at useful campaign scale, recover it reliably, and link it to sequence and function without adding a new bottleneck?”. In throughput terms, a rough architecture-specific pattern has emerged. Chamber and nanopen systems typically operate in the range of thousands to tens of thousands of cells but provide rich functional information per cell. Microwell systems sit in a moderate-throughput regime, often sacrificing absolute scale for imaging compatibility and repeated measurement. Microcapillary array and droplet-based workflows remain the throughput leaders, with studies describing screening scales of 10^5^ to 10^7^ cells per experiment, particularly when coupled to Fluorescence-Activated Droplet Sorting or hybrid droplet/FACS pipelines [33,35,47]. The importance of that scale is not merely statistical; it determines whether rare specificities or rare functional phenotypes can be found without strong pre-enrichment. Equally important has been integration with established laboratory infrastructure. A recurring lesson from the last decade is that technologies gain traction faster when they plug into workflows scientists already know. For example, converting single-cell microfluidic encapsulation into sortable particles compatible with conventional FACS removes one of the historic barriers to adoption of bespoke droplet platforms. Similarly, chamber-based nanopen systems gained momentum not just because they could assay single B cells, but because they combined confinement, imaging, export, and downstream sequencing in one commercial ecosystem. Another major aspect of scalability is data integration. As throughput has increased, the limiting factor has often shifted from physical screening to interpretation. Recent repertoire-profiling and machine-learning studies show that large single-cell datasets can be used to train predictive models of antigen specificity and B cell prioritisation, but only if experimental labels are sufficiently rich and well-linked to sequence [7,38]. This is pushing the field toward platforms that produce not just more cells, but more informative cells, cells with coupled measurements of secretion, specificity, transcriptional state, and clonotype identity. The net result is that scalability is no longer synonymous with raw cell count. In the current literature, the most valuable platforms are those that can scale in a workflow sense: they support large or meaningful cell numbers, integrate seamlessly with cell recovery and sequencing, and generate datasets that can be acted on computationally. That is the foundation of the more recent move toward closed-loop antibody discovery.

### 2.5. Emergence of Function-First Antibody Discovery

**Figure 3:**
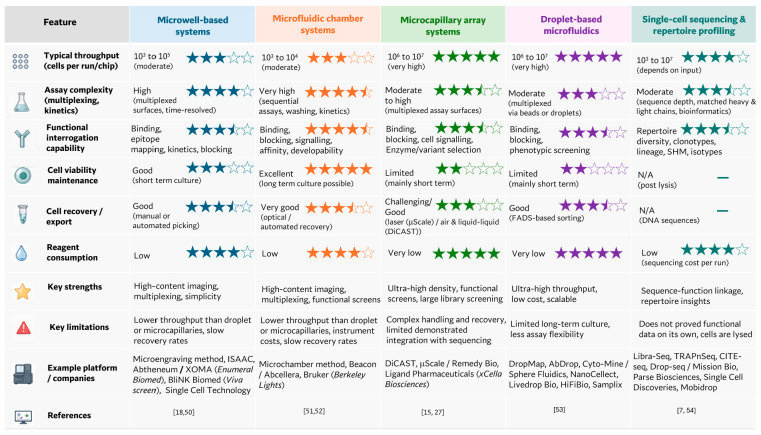
Comparison of single-cell analysis technologies for antibody discovery. The star-based rankings and associated attributes presented in this table are intended to reflect generalised characteristics of each class of single-cell microtool platform, rather than any specific individual technology. These evaluations and example platforms are not exhaustive and should not be interpreted as definitive comparisons. Individual platforms within each category may vary significantly in their capabilities and performance across different features [7,15,18,27,50,51,52,53,54].

A major conceptual change over the last 10 years (Figure 3) has been the move from binding-first to function-first discovery. Traditional workflows often used antigen binding as the primary gate and left biological function, neutralisation, receptor blockade, agonism, signalling modulation, or developability for later rounds [18,48]. Newer single-cell platforms increasingly collapse those stages, allowing biologically relevant behaviour to become the earliest selection criterion. Reviews of recent single-B-cell technologies emphasise that this is one of the defining shifts in the post-COVID era, particularly for infectious disease antibodies where neutralisation or blocking activity is more important than simple target recognition [32,38]. Microfluidic chambers and microcapillary arrays have been particularly influential here because they allow repeated and sequential assay steps on the same cell. These systems can measure secretion, binding, ligand blocking, and sometimes developability-relevant behaviour from a single secreting cell before export. Droplet systems are also increasingly function-capable, especially when reporter cells, capture beads, or hydrogel retention strategies are used.

A second defining trend is the growth of multiplexing. In practical terms, this means using panels of antigens, barcodes, fluorescent readouts, or repeated reagent cycles to derive multiple functional or specificity features from one cell. LIBRA-seq [40] showed the power of multiplexed specificity mapping at the sequencing level, while chamber and nanopen systems [24,49] extended multiplexing into phenotypic assays such as receptor blocking or epitope differentiation. The result is a shift from one-cell/one-readout workflows toward high-dimensional functional profiling, where each cell can be described by multiple attributes such as secretion, specificity, cross-reactivity, and molecular features. The practical consequence of function-first and multiplexed discovery has been a measurable compression of timelines. The clearest examples come from infectious-disease discovery, where single-cell screening platforms have enabled potent anti-viral antibodies to be identified in days to weeks rather than months [29]. The broader therapeutic impact is that these platforms reduce attrition by selecting better candidates earlier. Instead of carrying forward large numbers of binders into expensive downstream validation, function-first systems enrich directly for antibodies with desired biological behaviour. This can improve both speed and quality of lead generation, especially in areas such as infectious disease, oncology, and immunomodulation, where mechanism matters as much as specificity.

Collectively, the rapid evolution of single-cell microtools, droplet microfluidics, and sequencing-based platforms has generated an unprecedented volume of high-quality, multidimensional antibody data, encompassing sequence, specificity, functional activity, and cellular context. These datasets are now providing the critical foundation for the integration of computational and artificial intelligence (AI)-driven approaches into antibody discovery workflows [55,56,57]. In particular, the direct linkage of genotype to phenotype at scale enables the training of machine learning models capable of predicting antigen specificity, binding affinity, developability, and functional mechanisms from sequence alone. High-dimensional datasets derived from multiplexed and function-first screening platforms are especially valuable, as they move beyond binary binding classifications to capture nuanced biological activity, thereby enabling more accurate and generalisable predictive models. In this framework, single-cell technologies serve not only as discovery tools but also as data-generation engines, continuously refining predictive models and enabling iterative optimisation of antibody candidates.

## 3. Phase 2: Computational Analysis & Generative AI

### 3.1. Computational Repertoire Analysis

**Figure 4:**
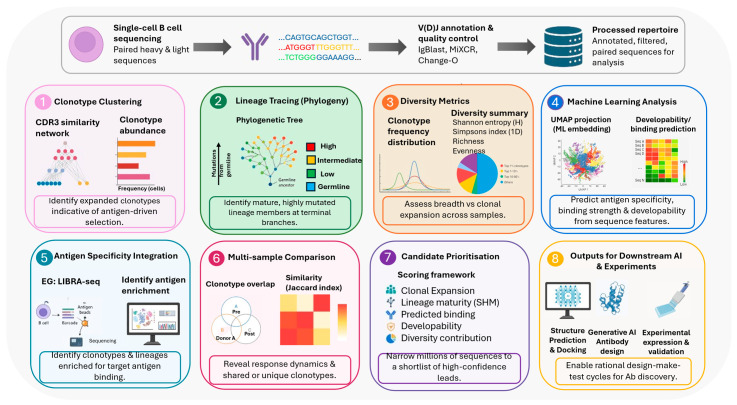
Computational repertoire analysis pipeline from antibody sequences to functional insights. This figure outlines the key computational steps used to transform raw single-cell antibody sequencing data into biologically meaningful insights. Following paired V_H_/V_L_ sequence acquisition, datasets undergo V(D)J annotation and quality control. Essential analytical steps involve (**1**) clonotype clustering to identify clonally expanded B cell populations, (**2**) lineage tracing to reconstruct somatic hypermutation and affinity pathways, (**3**) diversity metrics to quantify repertoire composition and clonal expansion, (**4**) machine learning analysis to identify patterns linked to antigen specificity, binding and developability, (**5**) antigen specificity integration to associate clonotypes with target antigen binding profiles, (**6**) multi-sample comparison to evaluate shared and unique clonotype dynamics across samples or conditions, (**7**) candidate prioritisation to rank high-confidence antibody leads based on expansion, maturation, binding prediction and developability features, and (**8**) downstream AI and experimental outputs enabling structural prediction, generative antibody design and experimental validation workflows.Icons were generated in Biorender.com.

Computational repertoire analysis (Figure 4) represents a foundational component of in silico antibody discovery. It enables the transformation of large-scale single-cell sequencing data sets into biologically meaningful insights that guide AI-driven design. Once single-cell RNA sequencing is carried out, the sequences of the paired heavy and light chain can be acquired. Computational methods can be applied to characterise the structure, diversity, and evolutionary dynamics of the B cell repertoire. For the selection of the best high-quality antibody, these analyses allow for the identification of enriched antigen-specific clones and understanding of affinity maturation pathways. Clonotype clustering is a central step in repertoire analysis. It involves clustering groups with antibody sequences based on shared V(D)J gene usage and high sequence similarities within the complementary determining region 3 (CDR3). The CDR3 region is highly variable and largely determines antigen specificity, making it a key feature for defining clonotypes [58]. Computational tools like IgBlast, MiXCR and Change-O allow the annotation of V(D)J segments and the grouping of sequences into clonally related families [59,60]. By identifying clonotypes that appear at a high frequency within a dataset, researchers can use antigen-driven selection. It is highly likely that these clones are a representation of B cells that have undergone proliferation in response to infection or immunisation. In studies of SARS-CoV-2, this approach was most notably used to rapidly identify sources of potent neutralising antibodies from expanded clonotypes [61].

Beyond clustering, lineage tracing provides a deeper insight into the evolutionary trajectories of antibody responses. Through reconstruction of phylogenetic trees of clonally related sequences, researchers can model the somatic hypermutation process and affinity maturation that occurs in germinal centres [62,63]. These lineage trees reveal how mutations accumulate over time and allow for the mature antibody variants with improved binding affinity to be identified. IgPhyML and partis are computational frameworks which use probabilistic models to find ancestral sequences and mutation pathways [63,64]. The sequences that lie at the terminal branches of the tree often have higher specificity and affinity. Lineage analysis is important as it allows for the prioritisation of these sequences. Within the immune system, some candidates may have undergone natural optimisation; applying this evolutionary perspective gives a rational basis for selecting these candidates. The quantification of diversity metrics is another important area of repertoire analysis. It focuses on the overall quality and structure of the antibody population. There are a few metrics commonly used in assessing repertoire diversity, like Simpson’s diversity index, Shannon entropy and clonotype frequency distributions [65]. A broad immune response is represented by high diversity; however, reduced diversity with dominant clonotypes suggests focused antigen-specific expansion. These metrics are particularly important when comparing conditions like healthy vs. diseased or pre- and post- immunisation repertoires. Diversity analysis in the antibody discovery process ensures that rare but potentially high-affinity binders are not overlooked. These computational repertoire analyses incorporate machine learning approaches to find higher-order patterns from sequence data. Unsupervised learning techniques, including clustering algorithms and dimensionality reduction methods such as UMAP or t-SNE, are used to visualise repertoire structure and identify distinct subpopulations of B cells [66]. These approaches enable the prediction of functional properties directly from sequence data, further refining candidate selection prior to structural modelling or experimental validation.

The integration of repertoire analysis with functional screening data further enhances its utility. Technologies such as LIBRA-seq link B-cell receptor sequences with antigen-binding information, allowing computational pipelines to directly associate specific clonotypes with target specificity [40]. This multimodal data integration enables more accurate identification of enriched binders and reduces reliance on indirect inference from sequence frequency alone. As a result, computational repertoire analysis becomes not just a descriptive tool, but a predictive engine that informs the entire antibody discovery pipeline. It aims to narrow the candidate search from millions of sequences to a high-confidence lead subset. By combining these approaches, researchers can prioritise candidates that are both structurally promising and biologically relevant. The selected antibodies can be passed on to the downstream stages, which include structure prediction, docking and generative AI optimisation. Repertoire analysis acts as an important bridge between raw sequencing data and AI-driven antibody engineering. This creates a more rational and data-driven antibody discovery process.

### 3.2. 3D Structure Prediction

**Figure 5:**
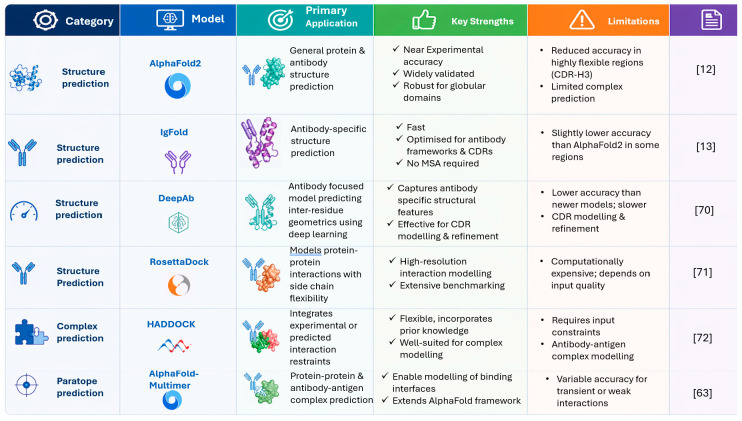
Summary of AI-driven structure prediction tools in antibody discovery [12,13,63,70,71,72].

Three-dimensional structure prediction has become a central pillar of in silico antibody discovery. It enables the transition from sequence-based analysis to structure-guided design. Following computational repertoire analysis, where candidate antibody sequences are prioritised, structural modelling provides critical insight into how antibodies physically interact. Antibody function is not only determined by their sequence composition but also by the precise spatial arrangement of residues within the binding interface. The recent advances in AI have transformed this stage by enabling rapid and highly accurate antibody structure prediction directly from sequence data. AlphaFold is at the forefront of these developments. This technology has demonstrated near accuracy for a wide range of proteins [12] and its architecture integrates evolutionary information from multiple sequence alignments (MSA) with learned spatial relationships between residues. AlphaFold uses Evoformer architecture to jointly embed evolutionary and pairwise features from the sequence data [12]. The Evoformer processes the primary amino acid sequence along with multiple sequence alignments of evolutionary-related proteins. This is followed by a structure module that treats the protein as a residue gas of independent rotations and translations. It models each amino acid as a separate entity in space, where each has a position (translation) and an orientation (rotation). Chain constraints enforced by proteins make it difficult to optimise but AlphaFold temporarily ignores the chain connectivity and allows all residues to move freely and simultaneously [12]. The AI model will then adjust the positions of atoms until the residues are in the correct orientation. It will feed this predicted structure back into itself, to continuously refine the structure until a final physically realistic structure is produced. This allows it to predict full-atom 3D structures alongside confidence metrics such as predicted Local Distance Difference Test (pLDDT) and predicted aligned error (PAE). AlphaFold provides a robust starting point for modelling framework regions and a majority of the antigen binding sites. Modelling antibodies is not without its challenges, especially in the highly variable CDR loops. CDR-H3 often exhibits significant conformational flexibility and is more difficult to predict accurately [67]. IgFold and DeepAb were developed to combat these issues; these are two specialised antibody-focused models established and specifically trained on antibody structural datasets [14]. More recent advancements in protein structure prediction have introduced all-atom and protein language model-based approaches that further expand the computational toolkit available for antibody modelling. AlphaFold3 represents a significant progression from AlphaFold2 by incorporating diffusion-based modelling strategies and improved handling of protein complexes, ligands, nucleic acids, and biomolecular interactions, enabling more comprehensive structural predictions beyond single protein folding [68]. Similarly, RFdiffusion and related all-atom diffusion frameworks have demonstrated improved flexibility in modelling dynamic structural features and molecular interactions [69]. In parallel, protein language model-based approaches such as ESMFold use large-scale sequence learning rather than reliance on multiple sequence alignments, enabling rapid structure prediction directly from amino acid sequence data [70]. The prediction of variable regions and CDR loop conformations has been significantly improved by these models, resulting in a more accurate representation of antigen binding sites. There has also been advanced efforts to model antigen–antibody complexes using models like AlphaFold-Multimer [71]. RosettaDock and HADDOCK are additional models which simulate binding interactions and refine predicted complexes [72,73]. To streamline these concepts and highlight the complementary roles of different tools, key platforms used in antibody structure prediction and docking are summarised in Figure 5.

The integration of these tools enables a comprehensive structural workflow, starting from single-chain folding to complex modelling and interaction refinement. These modern prediction frameworks provide quantitative confidence metrics. Researchers can assess the reliability of certain regions within predicted structures. This is particularly useful when assessing uncertain regions, like flexible loops and the identification of exposed hydrophobic patches or structurally unstable motifs, regions that may require further experimental validation. Structural models can be used to evaluate developability characteristics, such as stability, aggregation and solubility [74]. By identifying these issues early in the discovery process, computational pipelines can filter out problematic candidates before entering costly experimental stages. This contributes to a more efficient and rational antibody development process.

The role of structural prediction extends beyond characterisation. These models act as a critical foundation for the next stage of the pipeline; the structural information is leveraged to guide design. Features such as predicted binding interfaces, paratope residues, and epitope compatibility provide the necessary constraints for rational optimisation. Enabling the transition from analysing naturally occurring antibodies to engineering improved variants. This shift marks a key inflection point in in silico antibody discovery. Rather than selecting from existing sequences, computational approaches begin to generate and optimise new candidates within defined structural and functional boundaries. Advances in generative AI now make it possible to design antibody structures and sequences de novo, while maintaining compatibility with target binding geometries. When coupled with downstream evaluation methods including docking, binding prediction, and molecular dynamics simulations, these approaches enable a fully iterative, closed-loop optimisation process. Together, this progression from structure prediction to generative design represents the convergence of data-driven modelling and rational engineering, forming the basis of next-generation computational antibody discovery pipelines.

### 3.3. Generative AI Design—Molecular Docking and Dynamics Simulation

**Figure 6:**
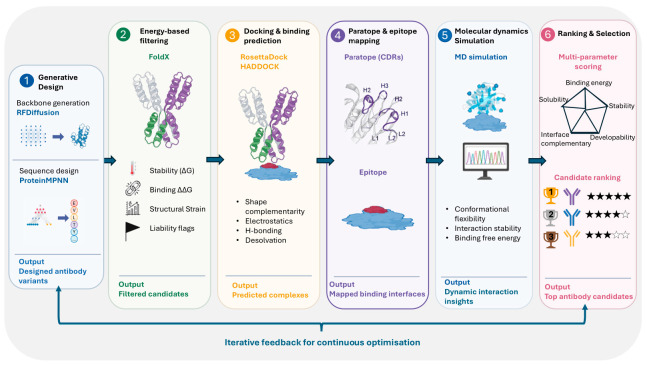
AI-driven antibody discovery and optimisation workflow. This figure summarises an integrated in silico pipeline for antibody design, combining generative modelling, structure prediction, and selection. Antibody candidates are generated using diffusion and protein language models. The weakest candidates are eliminated based on stability and binding energetics. Docking and binding predictions assess antibody–antigen interactions. This is followed by paratope/epitope mapping and molecular dynamics to evaluate binding interfaces and structural stability. Finally, top candidates are prioritised based on affinity and developability. Icons were generated in Biorender.com.

Recent advancements in generative AI have enabled the direct design of protein structures and sequences with desired functional properties (Figure 6). Models such as RFdiffusion represent a breakthrough in this space. The diffusion-based model generates protein backbones conditioned on structural constraints [69]. In the context of antibody discovery, RF diffusion can be used to design novel binding interfaces or scaffold CDR-regions that are geometrically compatible with the target epitope. It has been successfully applied to generate de novo protein binders with high shape complementarity and experimentally validated binding activity against predefined targets [69]. These models operate in 3D space applying precise control over features essential for antigen recognition. Sequence design is performed using models such as ProteinMPNN following on from backbone generation. This model optimises amino acid sequences to stabilise a given framework [75]. Structurally compatible and biologically plausible sequences are generated by graph-based neural networks. ProteinMPNN and RFdiffusion work together to form a powerful pipeline. Building on this framework, specialised adaptations such as RFdiffusion Antibody extend this approach by fine-tuning antigen–antibody complex structures to enable targeted CDR design [76]. In these models, the antibody framework and antigen are held fixed while noise is iteratively applied and removed from the CDR regions, allowing the generation of diverse backbone conformations that dock to predefined epitope “hotspot” residues. The resulting structures are subsequently paired with sequence design tools such as ProteinMPNN and refined using energy-based metrics (e.g., ΔΔG) and structure prediction models, enabling the identification of candidates with favourable binding interactions and structural stability [75]. This approach has been shown to generate novel VHH and scFv designs with accurate epitope targeting and experimentally validated binding, demonstrating atomic-level precision in antibody–antigen interface design. These models operate directly in 3D space, allowing precise control over structural features that are critical for antigen recognition and enabling the exploration of binding solutions beyond those accessible through natural immune repertoires. Complementing generative backbone and sequence design, physics-based energy evaluation tools such as FoldX provide a critical layer of validation. FoldX enables rapid estimation of folding stability and binding free energy changes (ΔΔG), allowing prioritisation of mutations that improve affinity while maintaining structural integrity. Recent studies have demonstrated its effectiveness in antibody optimisation pipelines, where it is used to refine and filter AI-generated candidates prior to more computationally intensive docking and simulation steps [77].

Following generative design, candidate antibodies must be evaluated for their ability to bind target antigens. This is achieved through molecular docking and binding prediction, which simulate antibody–antigen interactions and estimate binding affinity. Docking tools such as RosettaDock and HADDOCK are widely used to model the orientation and interaction of antibody variable regions with antigen epitopes [72,73]. The factors considered by these models are shape complementarity, electrostatics, and hydrogen bonding to predict energetically favourable binding conformations. AI-based docking approaches are being developed to improve both speed and accuracy, particularly for flexible antibody loops. A critical component of this stage is the identification of paratopes and epitopes, which define the interacting regions on the antibody and antigen, respectively. Computational tools can predict paratope residues directly from sequence or structure, highlighting key binding determinants within the CDR loops. Similarly, epitope prediction methods identify antigen surface regions most likely to be recognised by antibodies. Integrating these predictions with docking results enhances the accuracy of interaction modelling and provides mechanistic insight into binding specificity [78]. Molecular dynamics (MD) simulations are utilised to further refine candidate evaluation. They are employed to capture the dynamic behaviour of antibody–antigen complexes over time. Unlike static docking models, MD simulations provide a more realistic representation of binding interactions. They present the conformational flexibility, and thermal fluctuations. These simulations can reveal the stability of complexes and estimate binding free energies through methods such as MM/PBSA or free energy perturbation [79]. This dynamic perspective is particularly important for antibodies, where subtle conformational changes in CDR loops can significantly impact binding affinity.

The outputs of docking and simulation are integrated for candidate ranking using multiple criteria, including predicted binding energy, structural stability, interface complementarity, and developability metrics. Machine learning models increasingly combine these features into composite scoring functions, enabling more accurate prioritisation of lead candidates. This multi-parameter optimisation ensures that selected antibodies are not only high-affinity binders but also suitable for downstream therapeutic development. Importantly, this process is highly iterative and forms part of a closed-loop design cycle. Generative models propose new variants, which are evaluated through structural modelling and docking, with results fed back to guide further optimisation. This enables rapid exploration of sequence and structural space, significantly reducing optimisation timelines. Despite these advances, challenges remain, including accurately modelling conformational flexibility and limitations in current scoring functions and training data. Many AI models are highly dependent on the quality and diversity of available datasets. This can introduce bias and reduce generalisability across different antigen classes or antibody formats. Predictive models often perform best in well-represented protein targets but may struggle on rare epitopes, non-protein antigens or highly flexible binding interfaces. In addition, many deep learning frameworks operate as “black-box” systems, providing limited biological interpretability. This makes it difficult to rationalise why specific structures or sequences are prioritised. Nevertheless, continued improvements in AI methods and hybrid modelling approaches are rapidly enhancing the efficiency and reliability of in silico antibody discovery formats.

### 3.4. Computational Filtering: Binding Affinity and Developability Assessment

Computational filtering represents a critical downstream step in in silico antibody discovery. It allows the high-quality antibodies to be prioritised by integrating binding affinity predictions and key developability characteristics. Following the previous stages in the pipeline, numerous antibody candidates are produced. It is crucial to eliminate the low potential designs early to reduce the experimental burden and improve the efficiency of the pipeline. Computational filtering excludes the antibodies with the suboptimal biophysical properties. The primary focus of computational filtering is binding affinity evaluation. This reflects the specificity and strength of antibody–antigen interactions. Docking scores and energy calculations are structure-based approaches for estimating binding potential. These assess shape complementarity, hydrogen bonding and electrostatics [72]. Molecular dynamics-based free energy calculations further refine binding predictions by incorporating temporal interaction energetics, whereas machine learning models enable high-throughput prediction of binding directly from sequence or structural inputs [79,80]. The assessment of developability is important also. It determines whether a candidate is suitable for therapeutic use and can be manufactured at scale. The key properties of a candidate are stability, solubility, and low aggregation propensity. Computational tools analyse structural and sequence features to identify liabilities. These include charge distribution, hydrophobic patches and aggregation-prone motifs. There are platforms that integrate multiple metrics to flag high-risk candidates to enable efficient screening of large datasets. Therapeutic Antibody Profiler is an example of such a platform [81]. Despite its advantages, computational filtering remains limited by the accuracy of underlying models and input structures, particularly in flexible regions such as CDR loops. Developability predictions may not fully capture the diversity of AI-generated sequences. Predictive performance is constantly being improved by advances in hybrid AI and physics-based methods.

### 3.5. Integration of Single Cell and In Silico Pipelines: Closed-Loop Antibody Discovery

**Figure 7:**
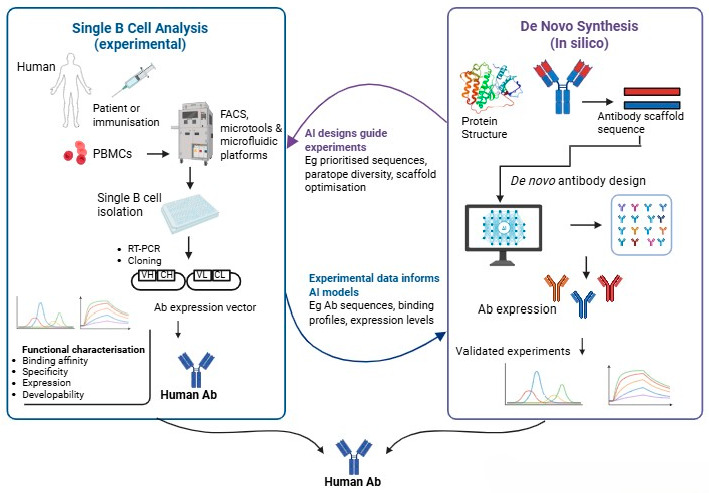
Schematic representing the importance of a feedback loop for closed-loop antibody discovery. The figure shows closed-loop framework linking experimental single-cell antibody discovery with in silico design and optimisation. On the experimental side (**left**), antigen-specific B-cells are isolated using platforms such as FACS, microtools and microfluidics. This is followed by paired V_H_/V_L_ sequencing, cloning and functional characterisation to generate high-resolution datasets connecting sequence, specificity and developability. These datasets inform the computational arm (**right**), where AI-driven models enable de novo antibody design, sequence optimisation and structural refinement. Experimental measurements, such as binding affinity, expression and stability are fed back into computational models to improve prediction accuracy. The AI-designed candidates guide the next rounds of experimental screening. The likelihood of identifying high-quality antibody leads is increased by moving from a linear process to this continuous feedback loop. Created in Biorender.

A significant shift from traditional workflows can be seen in the convergence of single-cell technologies with computational optimisation. High-resolution experimental data generated from single-cell platforms are coupled with machine learning and in silico design tools. This integrated framework creates a continuous cycle of prediction, validation and refinement (Figure 7). The iterative nature of this approach is a key advantage. The closed-loop system allows for the repeated cycles of optimisation in which computational predictions inform experimental validation. This newly generated data can be fed back into AI models. Candidate sequences with enhanced binding or developability characteristics can be proposed by these generative AI models. The sequences are subsequently synthesised using high-throughput expression systems such as mammalian display or CRISP-Cas9-mediated genome editing [82]. Functional screening assays evaluate candidates for binding affinity, specificity and biological activity. The resulting data are reintegrated into computational models allowing for continuous refinement. This accelerates the antibody discovery process, compressing cycles that traditionally required months or years into weeks. Several recent studies have demonstrated the practical impact of this integrated strategy. For example, the rapid identification of broadly neutralising antibodies against SARS-CoV-2 using LIBRA-seq illustrates how single-cell sequencing combined with antigen-specific screening can generate high quality datasets for computational analysis [42]. The synergy between experimental and computational approaches is further demonstrated through AI-driven optimisation of therapeutic antibodies such as trastuzumab, where deep learning models were used to improve binding affinity and developability profiles [83]. Similarly, machine learning-assisted affinity maturation approaches have enabled the generation of highly diverse sub-nanomolar affinity antibody libraries through iterative cycles of computational prediction and experimental validation [84]. Together, these studies highlight how high-throughput screening, sequencing, and AI-guided optimisation can function within closed-loop workflows, where experimental data continuously inform and refine computational models for antibody discovery [42,83,84]. Single-chain variable fragment optimisation using the Bayesian language model was successfully applied. This allowed for the exploration of sequence space while maintaining functional integrity [84]. These examples highlight the practical need of closed-loop antibody engineering frameworks. 

In a typical workflow, single-cell platforms identify antibody candidates. Paired V_H_-V_L_ regions are then sequenced and associated with functional data. Computational models analyse datasets to predict performance and uncover sequence–structure–function relationships. AI models can then generate optimised candidates for synthesis and experimental validation. Resulting data can then be fed back into this system, and further refinement can be carried out. Despite advances within AI-guided antibody discovery, there are still challenges in creating a closed-loop system. The quality and diversity of training data is a determining factor in the accuracy of computational models. Biases within experimental datasets influence predictive performance. Sophisticated modelling approaches and robust data standardisation are required for integration of heterogeneous data types. Another limitation is that computational predicitions do not always accurately reflect real-world biological behaviour, particularly for complex properties such as immunogenicity, developability and off-target interactions. Many advanced workflows require substantial computational infrastructure and specialist bioinformatics which may limit accessibility in smaller or primarily wet-lab-based research environments. As a result, experimental validation remains essential and continued advancements in hybrid physics-based and data-driven modelling approaches are necessary. This will improve the reliability, intrepretability and broader applicability of AI-assisted antibody discovery systems.

## 4. Phase 3: Selection and Experimental Validation

The third phase of modern antibody discovery involves the selection, experimental expression, and validation of in silico, predicted candidates, representing the critical interface between computational design and real-world biological performance [85]. Following model-driven prioritisation, whether based on predicted binding, developability, or functional activity, selected antibody sequences are synthesised, expressed recombinantly, and subjected to experimental assays to confirm their properties. These assays typically include antigen binding, e.g., ELISA [86], Flow Cytometry [87], Western blotting [88], Immunoprecipitation [89], SPR/BLI [90]); functional activity (e.g., neutralisation [91], receptor blocking [92], cell-based functional assays [93] and knockout cell lines [94]); and developability assessments such as stability, aggregation propensity, and expression yield [95]. Importantly, this phase serves not only to validate computational predictions but also to identify discrepancies between predicted and observed performance, which remain common due to the complexity of antibody structure–function relationships.

Crucially, the outcomes of experimental validation feed directly back into the computational pipeline, forming an iterative feedback loop that underpins closed-loop antibody discovery (Figure 7). High-quality datasets linking sequence, predicted properties, and experimentally measured outcomes are used to retrain and refine machine learning models, improving their predictive accuracy over successive cycles. This process enables models to better capture subtle determinants of antibody behaviour, including epitope specificity, conformational effects, and developability constraints that are difficult to encode explicitly. As more data is generated across diverse targets and formats, these systems evolve from predictive tools into adaptive learning frameworks, capable of generalisation across antibody classes and design objectives. This integration of in silico selection with experimental validation accelerates convergence toward optimal candidates and supports the transition of antibody discovery into a data-driven, self-improving engineering discipline. As a result, antibody discovery is transitioning toward a hybrid experimental–computational discipline, where in silico design, structure prediction, and generative modelling approaches are increasingly integrated with high-throughput single-cell platforms. This convergence is expected to accelerate the identification of high-quality therapeutic antibodies and ultimately enable more predictive, efficient, and scalable discovery pipelines.

### Limitations of AI-Driven Antibody Discovery in a Wet-Laboratory Settings

A major limitation of AI-driven antibody discovery and single-cell analysis is the level of computational expertise required to effectively implement and interpret these approaches. Although models such as AlphaFold and IgFold have become increasingly accessible, many wet-laboratory environments still lack the bioinformatics infrastructure, computational resources, and specialist knowledge needed for routine adoption. Successful use of these tools often requires experience in programming, machine learning, structural biology, and management of large sequencing datasets, creating a barrier for smaller academic laboratories without dedicated computational support. In addition, many AI workflows rely on high-performance computing resources such as GPUs and cloud-based systems, which may not be financially accessible in all research settings. Another important limitation is that many deep learning models function as “black-box” systems, providing limited biological interpretability and making troubleshooting difficult when predictions fail experimentally. Predictive performance can also be affected by biassed or low-quality training datasets, limiting model generalisability across different antigen classes. Many current AI pipelines still require manual curation, command-line execution, and integration of multiple software tools, slowing widespread adoption in standard molecular biology laboratories. Consequently, experimental validation remains essential, and continued improvements in user-friendly, interpretable, and hybrid physics-based AI approaches will be necessary to improve the broader applicability of AI-assisted antibody discovery systems.

## 5. Conclusions and Future Directions

The integration of single-cell microtools, high-throughput screening, sequencing, and computational design has delivered substantial benefits to antibody discovery such as a dramatic increase in speed, scale, and data richness, enabling the interrogation of millions of cells and the identification of high-quality candidates within weeks rather than months. Function-first screening and multiplexed assays improve candidate quality early in the pipeline, reducing downstream attrition. In parallel, the coupling of experimental platforms with sequencing and AI-driven tools enables data-informed decision making, allowing researchers to prioritise candidates based on a combination of sequence, specificity, function, and developability. Collectively, these advances are transforming antibody discovery into a more predictive, efficient, and scalable process, with clear advantages in areas requiring rapid response, such as emerging infectious diseases.

Despite these advances, several limitations remain. Many high-throughput platforms involve trade-offs between scale and assay complexity, with droplet systems offering exceptional throughput but more limited assay flexibility, while chamber-based systems provide rich functional data at lower scale. Integration across platforms, particularly linking functional screening with sequencing and downstream expression, can still be technically challenging. On the computational side, AI models are often constrained by data quality, bias, and limited generalisability, particularly when trained on datasets derived from specific targets or experimental conditions. These challenges become even more pronounced for antibody discovery when dealing with non-protein antigens, especially glycans and carbohydrates. In comparison with protein targets, there are relatively few well-characterised anti-glycan antibodies and antibody–glycan interacting datasets. This is restricting for the development of AI and machine learning models in the development of robust frameworks [96,97]. Carbohydrate epitopes often exhibit structural heterogeneity, conformational flexibility and weak immunogenicity which further complicates antibody discovery. Future AI-assisted approaches may help address these challenges through transfer learning, multimodal modelling, synthetic dataset generation, and improved glycan-aware structural prediction methods. Furthermore, accurately predicting complex properties such as developability, immunogenicity, and in vivo efficacy remains a significant challenge, reflecting the inherent complexity of antibody biology.

Looking forward, key challenges include improving standardisation and interoperability across experimental platforms, expanding high-quality datasets for model training, and developing more robust methods for integrating sequence, structural, and functional data. Enhancing the interpretability of AI models will also be critical for building confidence in their predictions and facilitating their adoption in regulated environments. Ultimately, continued progress will depend on the tight integration of experimental and computational workflows, enabling truly closed-loop discovery systems that are not only high-throughput but also adaptive, predictive, and broadly applicable across diverse therapeutic targets.

In summary, the emergence of function-first discovery has changed both architecture and the ambition of antibody discovery platforms. The latest systems do not merely identify antibodies; they characterise functional potential, link it to sequence, and do so at a speed and scale that directly influences therapeutic timelines. That shift is central to the modern landscape of single-cell antibody discovery coupled with the power of in silico-based discovery and optimisation.

## Data Availability

No new data were created or analyzed in this study.
